# Supplementary material for: Mechanisms governing the pioneering and redistribution capabilities of the non-classical pioneer PU.1
Source: Nat Commun. 2020 Jan 21;11:402. doi: 10.1038/s41467-019-13960-2 (PMC6972792; doi:10.1038/s41467-019-13960-2)
Supplement: Supplementary file 7 — Source data [file 41467_2019_13960_MOESM7_ESM.zip › Source_Data/FigureS3/FigureS3C_MotifsTALL1/knownResults.html]

/misc/data/analysis/project\_PU1/predictionTALL1/motifs/PU1\_Kmeans14.all.redhomer - Homer Known Motif Enrichment Results


# Homer Known Motif Enrichment Results (/misc/data/analysis/project\_PU1/predictionTALL1/motifs/PU1\_Kmeans14.all.redhomer)

Homer *de novo* Motif Results  
Gene Ontology Enrichment Results  
Known Motif Enrichment Results (txt file)  
Total Target Sequences = 42103, Total Background Sequences = 40943

|  |  |  |  |  |  |  |  |  |  |  |  |
| --- | --- | --- | --- | --- | --- | --- | --- | --- | --- | --- | --- |
| Rank | Motif | Name | P-value | log P-pvalue | q-value (Benjamini) | # Target Sequences with Motif | % of Targets Sequences with Motif | # Background Sequences with Motif | % of Background Sequences with Motif | Motif File | SVG |
| 1 | A G T C T G A C C T G A A G T C C G A T C G A T T A G C A G T C C G A T A C T G G T C A | 5-CCACTTCCTKM,BestGuess:ETS:RUNX(ETS,Runt)/Jurkat-RUNX1-ChIP-Seq(GSE17954)/Homer(0.867) | 1e-1422 | -3.275e+03 | 0.0000 | 11052.0 | 26.25% | 2526.2 | 6.17% | motif file (matrix) | svg |
| 2 | C T G A T A C G G A C T A C G T A C G T A G T C T G C A A G T C G C A T G C A T A G T C G A T C | 6-ASTTTCACTTCC,BestGuess:PU.1:IRF8(ETS:IRF)/pDC-Irf8-ChIP-Seq(GSE66899)/Homer(0.980) | 1e-364 | -8.388e+02 | 0.0000 | 3975.0 | 9.44% | 1148.3 | 2.80% | motif file (matrix) | svg |
| 3 | C A T G G C T A C T G A T C G A A G T C A G T C C T G A T A G C T C G A C G T A | 5-NWAACCACAN,BestGuess:RUNX1(Runt)/Jurkat-RUNX1-ChIP-Seq(GSE29180)/Homer(0.932) | 1e-86 | -1.995e+02 | 0.0000 | 4299.0 | 10.21% | 2632.2 | 6.43% | motif file (matrix) | svg |
| 4 | G T A C G A T C G C T A A T G C G A T C C G T A A C T G C T A G A C T G A T C G C A T G G A T C C T G A | 6-CCACYAGRGGGCA,BestGuess:CTCF(Zf)/CD4+-CTCF-ChIP-Seq(Barski\_et\_al.)/Homer(0.939) | 1e-76 | -1.768e+02 | 0.0000 | 924.0 | 2.19% | 282.7 | 0.69% | motif file (matrix) | svg |
| 5 | T C A G A G T C C T G A T C A G T G A C G C A T A C T G G A T C C A G T A T C G | 8-RCAGCWGYTG,BestGuess:Tcf12(bHLH)/GM12878-Tcf12-ChIP-Seq(GSE32465)/Homer(0.912) | 1e-37 | -8.647e+01 | 0.0000 | 5406.0 | 12.84% | 4092.9 | 10.00% | motif file (matrix) | svg |
| 6 | C A T G A G T C G A T C G T A C A G T C C T A G A G T C A G T C A G T C G A T C A G T C G A C T A G C T A G T C | 2-GCCCCGCCCCCTBC,BestGuess:SP2/MA0516.1/Jaspar(0.945) | 1e-33 | -7.683e+01 | 0.0000 | 1374.0 | 3.26% | 790.7 | 1.93% | motif file (matrix) | svg |
| 7 | C T A G G T A C T A C G A T G C G T C A A C G T T C A G A T G C A C T G G A T C G T C A | 11-GCGCATGCGCA,BestGuess:NRF1(NRF)/MCF7-NRF1-ChIP-Seq(Unpublished)/Homer(0.979) | 1e-13 | -3.192e+01 | 0.0000 | 243.0 | 0.58% | 99.0 | 0.24% | motif file (matrix) | svg |
| 8 | G A T C G A T C T C G A G C A T C T G A A C T G T G A C C T G A C G T A A G T C | 17-CCATRGCAAC,BestGuess:PB0055.1\_Rfx4\_1/Jaspar(0.881) | 1e-4 | -1.026e+01 | 0.0001 | 1024.0 | 2.43% | 828.4 | 2.02% | motif file (matrix) | svg |
| 9 | A G C T A T C G C T G A G A C T G C A T T C A G T C A G G A T C | 2-YGATTGGC,BestGuess:NFY(CCAAT)/Promoter/Homer(0.934) | 1e-4 | -9.748e+00 | 0.0001 | 3206.0 | 7.61% | 2832.5 | 6.92% | motif file (matrix) | svg |
| 10 | A G T C G T A C A T C G A G T C A G T C G C T A A G C T T G A C C G A T A C G T A C T G T C A G C G T A C T A G | 18-CCGCCATCTTGGAG,BestGuess:YY1(Zf)/Promoter/Homer(0.892) | 1e-3 | -7.960e+00 | 0.0005 | 53.0 | 0.13% | 22.7 | 0.06% | motif file (matrix) | svg |
| 11 | C T G A A G T C A G T C G T C A A G C T C T G A A C G T A C T G C T A G | 8-RCCATATGG,BestGuess:NEUROD2/MA0668.1/Jaspar(0.871) | 1e-2 | -6.221e+00 | 0.0023 | 729.0 | 1.73% | 605.3 | 1.48% | motif file (matrix) | svg |
| 12 | C T G A C G T A G T C A A C G T A C T G C G T A C T G A C T G A A T G C A C G T A C T G C T G A C G T A C G T A | 17-AAATGAAACTGAAA,BestGuess:IRF1/MA0050.2/Jaspar(0.858) | 1e-2 | -5.181e+00 | 0.0061 | 43.0 | 0.10% | 21.8 | 0.05% | motif file (matrix) | svg |
